# Family resilience for older patients requiring long-term care: a concept analysis

**DOI:** 10.3389/fpubh.2026.1962121

**Published:** 2026-10-22

**Authors:** Bahaerguli Abulaiti, Abudureheman Mijiti, Rongjing Xu, Hefang Wang, Yan Li, Shoumei Jia, Anni Wang

**Affiliations:** 1 Department of Nursing, Kashi Prefecture Second People’s Hospital, Kashi, Xinjiang, China; 2 School of Nursing, Fudan University, Shanghai, China; 3 Fudan University Interdisciplinary Innovation Team for Smart Eldercare and Social Support (FDU-iCARE), Shanghai, China; 4 China Research Center on Disability, Fudan University, Shanghai, China

**Keywords:** aging, concept analysis, family resilience, long-term care, older adults

## Abstract

**Aim:**

To analyse the concept of family resilience for older patients requiring long-term care. Design: Concept analysis.

**Methods:**

Walker and Avant’s eight-step concept analysis method was applied. Seven Chinese and English databases (PubMed, Web of Science, Embase, CNKI, Wanfang, Weipu and SinoMed) were searched from inception to 15 September 2026 for studies addressing family resilience in the context of older patients requiring long-term care. A total of 30 papers were included.

**Results:**

Four defining attributes were identified: (1) positive cognitive appraisal of adversity, (2) adaptive reorganization and problem solving, (3) supportive intergenerational interaction, and (4) proactive engagement with external resources. The antecedents were (1) health status of the older adult and the caregiver, (2) socio-demographic characteristics of family members, (3) disease cognition, and (4) family resources. The consequences were (1) reduced psychological distress and improved mental health, (2) improved quality of care and patient outcomes, (3) enhanced family adaptation and restored functioning, and (4) family growth, that is, a rebuilt and stronger family.

**Conclusion:**

The analysis yielded a preliminary conceptual model in which family resilience for older patients requiring long-term care is understood as a recursive, dynamic family-level process. A clearly defined concept provides a theoretical basis for developing population-specific assessment tools and family-level psychosocial interventions, and underscores the public mental health relevance of supporting families engaged in long-term care. No Patient or Public Contribution.

## 1 Introduction

Advancements in medical technology have increased life expectancy, leading to a rapid rise in population aging globally, particularly in China. This trend has intensified the chronic health challenges associated with aging (1). By the end of 2025, China had 323.38 million people aged 60 and above, accounting for 23.0% of the total population. Nationwide, approximately 450 million individuals were living with chronic diseases, which accounted for 88.5% of all deaths. Long-term care insurance covered 308.55 million enrollees, with 1.93 million disabled individuals receiving care benefits (2). Owing to the prevalence of multiple, long-duration, recurrent diseases with poor prognoses, older individuals require sustained long-term family care. Presently, and in the foreseeable future, nearly 90% of disabled older adults continue to depend on family support. Long-term care for the majority of older adults is mainly borne by family members, and families face substantial financial and caregiving burdens that place the well-being of the whole family at risk. With the pressures of modern life and changes in family structure, adult children are increasingly sandwiched between childcare and eldercare. Older adults therefore receive less companionship and less emotional support, while community resources have only partly compensated. Intergenerational support is weakening, and their emotional needs are going unmet (3). Older adults who lack the interaction of family members, especially their children, may experience persistent loneliness in later life, leading to more serious mental and physical health problems (4). Even when caregivers give up their jobs to care for an older family member, the family’s income decreases and the caregiver’s sense of self-efficacy declines, placing a heavy burden on the entire family. This often forces changes in the family system in terms of role relationships among family members, family lifestyles, and the prioritization of family values (5). A family under sustained stress, such as illness or financial hardship, may experience cognitive, emotional, social or instrumental imbalances that disrupt family functioning.

Family resilience, a key facilitator within family dynamics, necessitates the strategic utilization and mobilization of resources, enabling adaptive responses to emerging challenges. Families with high levels of resilience can identify and enhance family strengths, integrating internal and external resources to promote effective coping in adversity and stress (6). The concept of family resilience is increasingly capturing the attention of scholars worldwide, highlighting its role in structuring experiences and emotions and enhancing interpersonal communication (7). Research on family resilience has primarily targeted children, adolescents and cancer patients, revealing population-specific attributes. The meaning of family resilience for older patients requiring long-term care remains unclear, with definitions frequently derived from other disciplines or populations, thus diluting the concept’s clarity. Concept analysis can help to reduce ambiguity, clarify definitions and ensure the accurate use of concepts in nursing theory and research (7). Previous work in this field includes concept analyses of individual caregiver resilience. Yet caregiving is fundamentally a family endeavor: families provide the bulk of long-term care, and depression, anxiety, and chronic stress are highly prevalent among caregivers. Family resilience may therefore constitute a family-level protective factor and a promising target for community-based prevention (8, 9). This study is dedicated to refining the definition of family resilience for older patients requiring long-term care, exploring its multifaceted nature, and informing the development of targeted assessment tools and intervention strategies.

## 2 Materials and methods

### 2.1 Study design

This study adopted the concept analysis method of Walker and Avant (10), which proceeds in eight steps: (1) select a concept; (2) determine the aims and purposes of the analysis; (3) identify all uses of the concept that can be discovered; (4) determine the defining attributes; (5) construct a model case; (6) construct borderline, related and contrary cases; (7) identify antecedents and consequences; and (8) define empirical referents. The Walker and Avant method was applied consistently throughout the manuscript; no other concept-analysis tradition was combined with it.

### 2.2 Operational definitions

Two operational definitions guided study eligibility. First, consistent with the World Health Organization and with Chinese gerontological practice, “older adults” were defined as individuals aged 60 years or older (11). Second, “requiring long-term care” was operationalized as older adults with chronic disease, disability or functional dependency—operationalized as impairment in activities of daily living (ADL) or instrumental activities of daily living (IADL)—who require sustained assistance with daily living and/or health management for at least 6 months, delivered predominantly by family members in home or community settings (2, 12). Studies focusing exclusively on family caregivers were eligible when the care recipient met the above definition, because family resilience is expressed at the family level and caregiver reports capture family-level processes; studies of individual caregiver resilience without any family-level focus were excluded.

### 2.3 Data sources and search strategy

A comprehensive literature search was conducted to capture the breadth of the concept across both Western and Eastern healthcare contexts. Seven electronic databases were searched from inception to 15 September 2026: PubMed, Web of Science, Embase, China National Knowledge Infrastructure (CNKI), Wanfang, Weipu and SinoMed. Medical Subject Heading (MeSH) terms and free-text words were combined using Boolean operators, including “family resilien*,” “family strengt*,” “long-term care,” “family caregiv*,” “home care,” “chronic diseas*,” “mental disease,” “older*,” “aged*” and “geriatric”; database-specific syntax, controlled vocabulary and field restrictions were adapted for each database. The full PubMed strategy is presented in Table 1, and the database-specific strategies for Web of Science and Embase are presented in Supplementary Table 1. The Chinese-database search strategies are available from the authors upon request.

**Table 1:** Full search strategy (PubMed example).

Set	Search terms
#1	“Family Resilience”[MeSH Terms] OR “family resilien*”[Title/Abstract] OR “family strengt*”[Title/Abstract] OR “family adaptation”[Title/Abstract] OR “family coping”[Title/Abstract]
#2	“Long-Term Care”[MeSH Terms] OR “long-term care”[Title/Abstract] OR “long term care”[Title/Abstract] OR “family caregiv*”[Title/Abstract] OR “informal care*”[Title/Abstract] OR “Home Care Services”[MeSH Terms] OR “home care”[Title/Abstract]
#3	“Aged”[MeSH Terms] OR “older*”[Title/Abstract] OR “aged”[Title/Abstract] OR “elderly”[Title/Abstract] OR “geriatric”[Title/Abstract] OR “Chronic Disease”[MeSH Terms] OR “chronic diseas*”[Title/Abstract] OR “Disabled Persons”[MeSH Terms] OR “disab*”[Title/Abstract]
#4	#1 AND #2 AND #3 (from database inception to 15 September 2026; language: English)

Inclusion criteria: (1) the study focused on family resilience or closely related family-level adaptive constructs; (2) the study population comprised older adults requiring long-term care as operationally defined above and/or their family members; (3) the content addressed the concept of family resilience, its influencing factors, assessment tools, or adaptation processes; and (4) the article was published in Chinese or English with an accessible full text. Exclusion criteria: studies addressing only individual-level resilience, conference abstracts lacking critical information, and inaccessible full texts. Concept analyses and instrument-validation studies were eligible when they directly informed concept uses, conceptual boundaries, or empirical referents, even if their samples were not restricted to older adults requiring long-term care.

### 2.4 Screening and data extraction

To ensure the scientific soundness and reproducibility of this concept analysis, two reviewers (BA and RX) independently screened titles and abstracts against the eligibility criteria, and the same two reviewers then independently assessed the full texts of potentially eligible reports. Data were extracted independently by the two reviewers using a standardized form covering authors, year, country, study design, population, definitions or uses of family resilience, candidate attributes, antecedents, consequences, and assessment instruments. At both the screening and extraction stages, disagreements were resolved through discussion, and when consensus could not be reached, a third senior reviewer (AW) adjudicated. Ultimately, 30 studies were included; the majority of empirical studies were qualitative or cross-sectional in design. The study selection process is shown in Figure 1.

**Figure 1:**
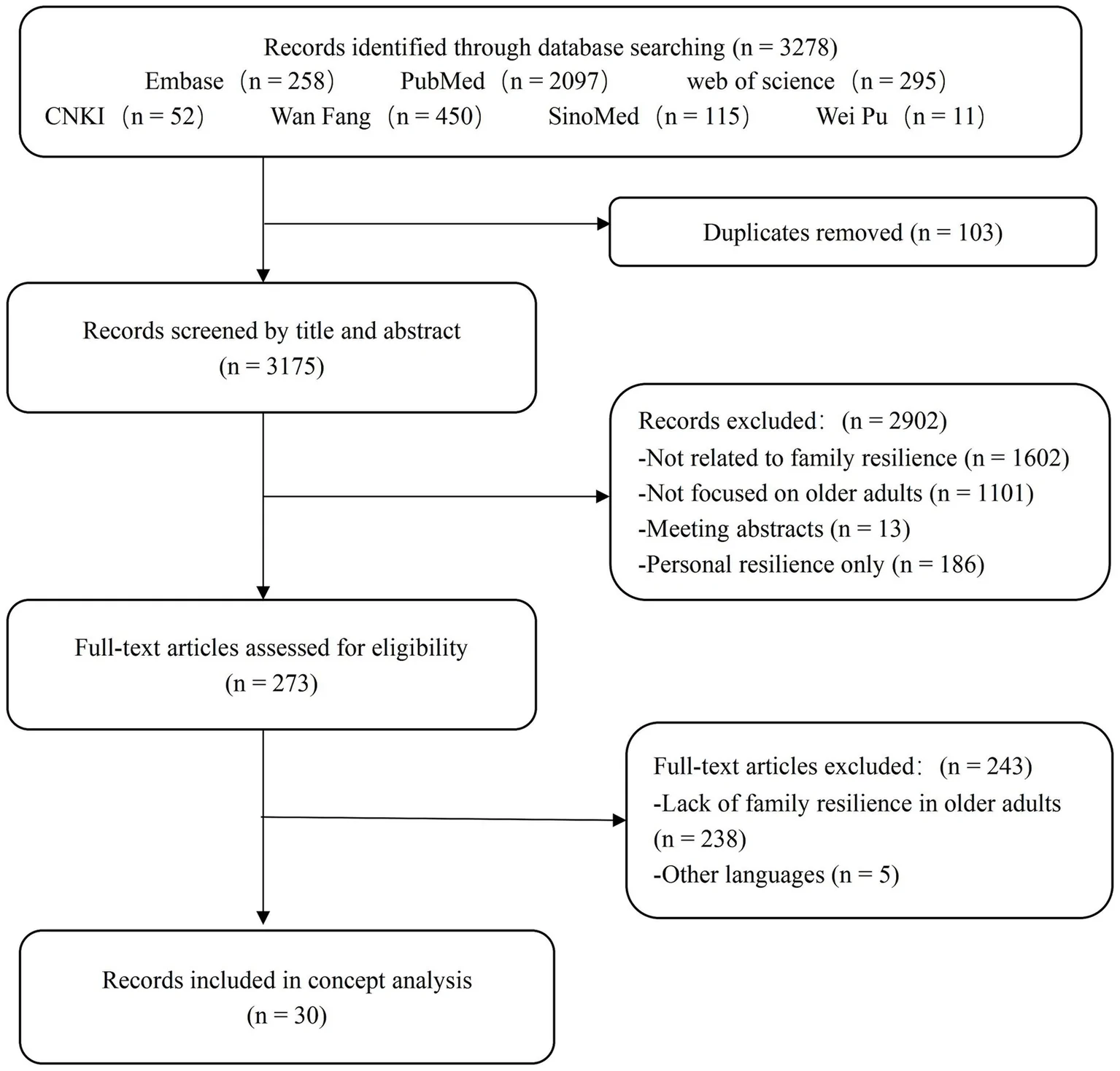
Flow diagram of the study selection process.

### 2.5 Method of analysis

Data analysis followed the eight-step framework established by Walker and Avant (10). Thematic analysis was employed to synthesize the extracted text into clusters representing attributes, antecedents and consequences. To classify each construct, an explicit temporal-logical criterion was applied: antecedents are conditions that exist prior to and shape the family’s adaptive response; defining attributes are the characteristics that constitute the concept itself when it is enacted; and consequences are states that follow from the enactment of the concept. A source-by-category analytical matrix documenting how each antecedent, attribute and consequence was derived from the included literature is presented in Table 2, which identifies each of the 30 included studies by reference number, authorship, country, design and population, so that every classification reported below can be traced to the eligible evidence. Similar concepts were contrasted to delineate conceptual boundaries. Model, borderline and contrary cases were constructed as hypothetical composite cases synthesized from the included literature to illustrate the full presence, partial presence and absence of the defining attributes, respectively; no human-participant data were collected for this study. A category-by-row view of the same evidence is provided in Supplementary Table 2.

**Table 2:** Characteristics of the 30 included studies and their detailed contribution to the concept analysis.

Study (reference)	Country	Design	Population/context	Attributes	Antecedent	Outcome
Muñoz-Cruz et al., 2024 (5)	Spain	Systematic review and meta-analysis	Family carers of dependent older people	—	Symptoms and self-care capacity (B1)	—
Zhang W et al., 2022 (6)	China	Qualitative (family strengths)	Families of first-episode stroke patients	Openness and positive family beliefs (A1); flexibility (A2); open communication (A3)	Symptom-management knowledge and coping repertoire (B3); family cohesion (B4)	—
Oh and Chang, 2014 (29)	South Korea	Concept analysis (Rodgers)	Family resilience literature (38 studies)	Collective confidence and positive life view (A1)	—	Acceptance, changed life perspectives, and reinforced resilient properties (C4)
Abulaiti B et al., 2022 (18)	China	Qualitative study	Home-dwelling older adults with disabilities and their family caregivers	Adjustment of family beliefs (A1); changes in family patterns and multichannel resource use (A2, A4); improvement of family communication (A3)	Disability severity and caregiver age/poor health (B1); disease/care knowledge (B3); family resources (B4)	Family adaptation to sustained care stress (C3)
Zhou and Wang, 2021 (19)	China	Cross-sectional	Homebound disabled older adults	—	Symptoms (B1); family type/structure, relationships, and functioning (B4)	Resource integration promoting health (C2)
Yan and Li, 2021 (17)	China	Concept analysis	Family resilience of cancer patients	Proactive and positive response to cancer-related stress (A1); mobilization of family strengths and restoration of balance (A2); use of internal and external resources (A4)	Cancer diagnosis, treatment, recurrence/metastasis, and disease course (B1); understanding and appraisal of disease (B3); family resources (B4)	Improved psychological and health behaviors (C1); resource integration, cooperation, and restored functioning (C3); positive development of the family (C4)
Lin et al., 2024 (26)	China	Cross-sectional (dyadic)	Older adults with chronic illness and caregivers	Motivation (A1); collective problem solving (A2); open communication (A3); accessing services (A4)	Symptoms (B1)	Improved care and patient quality of life (C2)
Jin et al., 2022 (34)	China	Cross-sectional	Older adults receiving home care	Emotional expression (A3); accessing services (A4)	Symptoms, disease duration/type, caregiving length, and caregiver health (B1); age and residence (B2); number of members, cohesion, faith/spirituality, economy, and social support (B4)	Return to normalcy (C3); family growth (C4)
Zhou J et al., 2022 (30)	China	Cross-sectional	Home-disabled older adults (Xinjiang)	Role reallocation and coordination (A2); mutual care, understanding, and coordination (A3); accessing services (A4)	Disease duration/type (B1); number of family members (B4)	—
Smith and Shaw, 2017 (42)	United Kingdom	Interpretative phenomenological analysis	Families living with Parkinson’s disease	—	Disease awareness (B3)	—
Nissen et al., 2016 (31)	United States	Cross-sectional	Caregivers of advanced cancer patients	Collective problem solving (A2); open communication (A3)	Symptoms, disease duration/type, and future uncertainty (B1); family functioning and cohesion (B4)	—
Yan and Zhang, 2017 (24)	China	Cross-sectional	Older patients with cerebral infarction	Encouragement, acceptance, responsibility, and motivation (A1); family control (A2)	Symptom-management knowledge and coping repertoire (B3); gender/communication (B2)	Reduced negative emotions and decreased anxiety/depression (C1)
Zhang et al., 2022 (25)	China	Cross-sectional	Home-disabled older adults (Xinjiang)	Encouragement (A1); role reallocation, coordination, and collective problem solving (A2); mutual care, understanding, coordination, and open communication (A3)	Age (B2); disease awareness (B3); family type/structure, relationships, and functioning (B4)	Symptom management and self-care (C2); reorganization, return to normalcy, and healthy development (C3)
Yu et al., 2022 (28)	China	Cross-sectional	Stroke family caregivers	Positive outlook and meaning-making (A1); flexibility (A2)	Disease duration/type (B1); gender and education (B2); family relationships, functioning, faith/spirituality, and economy (B4)	—
Deist and Greeff, 2017 (22)	South Africa	Qualitative	Families living with a parent with dementia	Optimism, acceptance, and motivation (A1); seeking and utilizing social support (A4)	Disease awareness (B3)	—
Ye et al., 2021 (21)	China	Cross-sectional	Caregivers of first-ever stroke patients	Perseverance, hope, motivation, and self-efficacy (A1); role reallocation and coordination (A2); mutual care and coordination (A3); accessing services (A4)	Symptom-management knowledge (B3); available social support (B4)	Reduced distress (C1); caregiving skills and disease knowledge (C4)
West et al., 2011 (35)	Australia	Conceptual model	Families living with chronic pain	Emotional expression (A3); seeking support outside the family (A4)	Faith and spirituality (B4)	—
Dunbar et al., 2008 (27)	United States	Cross-sectional	Families of patients with heart failure	Self-efficacy (A1); collective problem solving (A2); mutual care, coordination, and open communication (A3); seeking and utilizing social support (A4)	Family functioning (B4)	Symptom management, self-care, and treatment adherence (C2)
Sanchez-Teruel et al., 2022 (23)	Spain	Cross-sectional	Dementia caregivers during COVID-19	Hope and self-efficacy (A1); collective problem solving (A2); open communication and emotional expression (A3); seeking/utilizing social support and information (A4)	—	Well-being and reduced distress (C1)
Zhang M et al., 2021 (33)	China	Cross-sectional	Hospitalized stroke patients	Open communication (A3)	Disease awareness and perceived stress (B3)	—
Moral-Fernandez et al., 2018 (38)	Spain	Qualitative metasynthesis	Onset of caring for a dependent older family member	Information seeking and proactive coping (A4)	—	—
Zhang and Li, 2024 (39)	China	Grounded theory	Disabled older adults	—	—	Reorganization and return to normalcy (C3); family growth (C4)
Yang and Song, 2025 (40)	South Korea	Qualitative meta-synthesis	Families providing home-based dementia care	Adaptive reorganization and problem solving (A2); supportive family interaction (A3)	Disease cognition (B3); family resources (B4)	Enhanced family adaptation (C3); family growth (C4)
Kim et al., 2019 (32)	South Korea	Quasi-experimental intervention (FREP)	Families of older adults with dementia	Adaptive family organization and problem solving (A2); supportive family communication and cooperation (A3)	—	Improved family adaptation (C3)
Lv et al., 2026 (37)	China	Longitudinal (three-wave)	Dementia caregivers in community settings	Positive cognitive appraisal (A1); supportive intergenerational interaction (A3)	Health status (B1); family resources (B4)	Reduced psychological distress (C1)
Jia et al., 2025 (36)	China	Cross-sectional	Family caregivers of people with dementia	Supportive family interaction (A3)	—	Reduced caregiver burden and psychological distress (C1)
Wang and Lu, 2022 (49)	China	Psychometric validation	Community residents (WFRQ-C)	— (empirical referent)		
Zhang X et al., 2023 (43)	China	Psychometric validation	566 disabled older adults and caregivers (WFRQ-CE)	— (empirical referent)		
Li YL et al., 2016 (41)	China	Psychometric validation	Shortened Chinese FRAS	— (empirical referent)		
Liu Y et al., 2014 (55)	China	Psychometric validation	FHI Chinese version	— (empirical referent)		

A1–A4 = defining attributes; B1–B4 = antecedents; C1–C4 = consequences. A1 positive cognitive appraisal of adversity; A2 adaptive reorganization and problem solving; A3 supportive intergenerational interaction; A4 proactive engagement with external resources. B1 health status of the older adult and the caregiver; B2 socio-demographic characteristics of family members; B3 disease cognition; B4 family resources. C1 reduced psychological distress and improved mental health; C2 improved quality of care and patient outcomes; C3 enhanced family adaptation and restored functioning; C4 family growth: a rebuilt and stronger family.

## 3 Results

### 3.1 Uses of the concept and its development

Originating from early psychiatric studies, resilience was initially examined through qualitative analyses in children, revealing that even in similar environments individual resilience can vary substantially (13). Initially perceived as a trait, resilience has been reconceptualized in contemporary research as a dynamic and modifiable process. The focus on family resilience began with Patterson and McCubbin (14), who identified that families possess capabilities for repair and growth, with the strengths of individuals and relationships within families intensifying through collaboration in response to crises or enduring stress. Current research on family resilience focuses mainly on children, adolescents, and people with chronic illness and cancer.

The concept of family resilience lacks standardization and can be broadly categorized into four perspectives. Table 3 details these diverse interpretations across the international literature. Taken together, these perspectives indicate that family resilience is regarded as a positive process rather than a static trait, although the literature does not always clearly indicate whether resilience is a transformational process or the force necessary for that process—an ambiguity evident in Walsh’s (15) definition. In summary, family resilience is viewed as a characteristic, process, or outcome of a holistic response to stress and problem solving—a supportive force for family functioning through which families self-regulate to achieve better adaptation. Scholars have analysed the concept in cancer populations to clarify its operational definition (16, 17). Within the geriatric literature, related perspectives have been offered by earlier studies of families in long-term care (18–20). However, no concept analysis to date has articulated family resilience as a family-level construct among older patients requiring long-term care. Regardless of the definition, three core elements recur: viewing the family as a whole; the family experiencing stressful situations; and, even in the face of stress, the family adapting more quickly by working together.

**Table 3:** Overview of the development of the concept of family resilience.

Perspective	Reference	Definition
Trait/property	McCubbin and McCubbin, 1988 (61)	Characteristics, dimensions and properties of families that enable them to resist disruption in the face of change and to adapt in the face of crisis situations
	Hawley and DeHaan, 1996 (62)	The ability of families to coalesce and respond positively to life’s challenges
	Patterson, 1995 (63)	The family’s ability to mobilize strengths actively when family life is threatened by stressful events or crises
	Walsh, 1996 (15)	The adaptive capacity of families to maintain equilibrium in the face of crisis, and the potential for activating flexibility, problem solving and resource mobilization within the family
	Oh and Chang, 2014 (29)	The family’s capabilities to regain its psychological and functional integrity after adversity
	Wang et al., 2021 (16)	The good qualities of the family and its members, close family relationships, high family support and interaction with the outside world
	Zhou and Wang, 2021 (19)	When families face adversity or crisis with heavy caregiving pressure, the ability to assist family members in overcoming adversity through the initiative of family members
Process	Hawley and DeHaan, 1996 (62)	Family resilience develops over time; resilient families respond positively to adversity in their own unique ways and with their own resources, influenced by the interaction of environment, developmental level, and risk and protective factors
	Oswald, 2002 (64)	Emphasizes the relational nature of the family as a whole unit and the relational processes that contribute to the survival, and even growth, of the family in the face of stressors
	Lee et al., 2004 (58)	An enduring force that leads a family to change its functioning dynamics in order to solve the stress-related problems it encounters
	Walsh, 2003 (65)	The potential for personal and relational transformation and growth that develops in the face of adversity and enables families to overcome it
	Walsh, 1996 (15)	Viewing the family as a functional unit, family resilience is the ability to cope effectively with crises and stresses from within or outside the family, to recover and become stronger, and to gain access to more psychosocial resources
Outcome	Black and Lobo, 2008 (66)	Families utilize a variety of internal and external resources and strengths to cope with stress and adversity as they respond, achieving positive adaptation
	Dai, 2008 (53)	Not only the qualities that families possess or learn over time, but also the process of coping with and adapting to adversity as a whole functional unit, comprising multilevel and multidimensional factors that enhance the family’s ability to cope with and adapt to stress and adversity
Integrative	Henry et al., 2015 (67)	Consisting of an ecosystem, a family adaptation system and a family contextual meaning system, family resilience is a dynamic system of family interaction with the external environment in the face of stress, covering both the process and the outcome of adaptation
	Wang et al., 2018 (68)	The positive patterns of behaviour and the ability of individuals and families to maintain normal functioning in the face of stressful situations or adversity, which the family as a whole confronts and resists together, ensuring the restoration of normalcy or the maintenance of overall family functioning
	Yan and Li, 2021 (17)	When a family member is diagnosed with cancer, the family—as an independent functional whole—responds proactively to pressure to achieve adaptation, mobilizing internal and external resources and family strengths, displaying positive family characteristics and development, and seeking to restore balance and harmony

### 3.2 Defining attributes

Defining attributes are the characteristics, elements or components that appear most consistently when a concept occurs and that allow the concept to be distinguished from similar concepts (7). Applying the temporal-logical criterion, constructs that describe the family’s enacted adaptive response itself were classified as attributes, whereas pre-existing conditions and resources were classified as antecedents and subsequent changes as consequences. On this basis, four defining attributes of family resilience for older patients requiring long-term care were identified.

#### 3.2.1 Positive cognitive appraisal of adversity

This attribute refers to the shared tendency of family members to appraise the caregiving situation in a positive, meaningful and hopeful way. Family members exhibit perseverance (21), optimism (22) and hope (21, 23), together with openness (6), encouragement (24, 25), acceptance (22, 24), a sense of responsibility (24), motivation (21, 22, 24, 26) and high self-efficacy (21, 23, 27). At the family level, these individual dispositions converge into shared positive beliefs through which the family makes sense of the illness and caregiving, maintains a positive outlook, and mobilizes internal psychological resources to tackle caregiving challenges, thereby fostering positive experiences (18, 28). Such collective confidence and positive life views have likewise been identified as core dimensions of family resilience in broader populations (29).

#### 3.2.2 Adaptive reorganization and problem solving

This attribute refers to the family’s capacity to reorganize itself in response to caregiving demands. It encompasses family flexibility (6, 18, 28), the reallocation and coordination of caregiving roles among family members (21, 25, 30), collective problem solving (18, 23, 25–27, 31), and a sense of family control (24, 32). Whereas a repertoire of coping resources available to the family is treated as an antecedent, the enactment of flexible reorganization and joint problem solving in the face of concrete caregiving difficulties is a defining manifestation of family resilience itself: families with high resilience actively adjust their organizational patterns and work through problems together rather than avoiding them.

#### 3.2.3 Supportive intergenerational interactions within the family

This attribute refers to the reciprocal interactions through which family members care for one another, communicate emotionally and provide family support in daily life. The more resilient the family, the better its members are at communicating openly (6, 23, 25–27, 31, 33) and expressing their feelings and needs (18, 23, 34, 35), which strengthens mutual bonds and facilitates emotional exchange and understanding. In addition, family members support each other in caring for the older person (21, 25, 27, 30, 36), and show mutual understanding (25, 30) and coordination (21, 25, 27, 30). Open intergenerational communication acts as the “lubricant” of family resilience: it improves family relationships, solidarity and collaboration, and enables families to address current challenges together (25). In well-adapted families, intergenerational interaction in communication, emotional expression and mutual care plays a central role in coping with long-term care, and families consolidate their cohesion through reciprocal interaction to solve life problems jointly (37).

#### 3.2.4 Proactive engagement with external resources

This attribute refers to the family’s active interaction with its surrounding environment, including seeking and utilizing social support and resources (22, 23, 27), actively seeking information (23, 38), maintaining good social relationships, and accessing and using available services (21, 26, 30, 34). Maintaining good social relationships facilitates access to information, support and resources and improves quality of life and well-being. In East Asian societies such as China, family networks are usually strong, but under particular circumstances support must also be sought from outside the family (18, 35). Families that proactively seek ways to cope with caregiving stress—confiding in friends and relatives, participating in social activities, seeking spiritual or faith-based support, consulting other families facing similar problems, and requesting help from the community—demonstrate better family resilience (38).

### 3.3 Antecedents and consequences

#### 3.3.1 Antecedents

Antecedents are the preconditions that exist before the concept occurs. The antecedents of family resilience for older patients requiring long-term care included the health status of the older adult and the caregiver, socio-demographic characteristics of family members, disease cognition, and family resources. Constructs such as family cohesion, family functioning, spirituality and available social support were classified here, as pre-existing resources, rather than as defining attributes, because they describe conditions that precede and shape the family’s adaptive response rather than the response itself.

Health Status of the Older Adult and the Caregiver. This includes the older adult’s self-care capacity, symptoms (5, 19, 26, 31, 34), disease duration and disease type (18, 36, 39–41), future uncertainty (31), the caregiver’s health status (18, 19, 34, 37), and length of caregiving (34). Disease type, progression and symptoms alter older individuals’ self-care abilities and lifestyles, consequently affecting the family negatively. Worsening conditions necessitate continuous care, imposing psychological strains such as depression, anxiety and despair on caregivers, thereby heightening the caregiving burden and influencing family resilience (19). Good caregiver health and shorter caregiving hours help families deal positively with the stresses associated with long-term care (19). Learning to focus on the present amid uncertainty also facilitates family adaptation to the fear associated with an uncertain prognosis (42).

Socio-Demographic Characteristics of Family Members. This includes age (25, 34), gender (28), education level (41), and residential status (34). Although men and women exhibit similar levels of resilience, women tend to communicate more readily (24), are more sensitive to negative emotions (28), and frequently shoulder multiple caregiving roles. The current predominance of women as family carers further increases their caregiving burden (5, 36). Caregivers with higher educational attainment have access to more psychosocial resources, generate more positive cognitions and behaviors, and can learn about the disease through various channels, which is conducive to maintaining a high level of family resilience (28).

Disease Cognition. This comprises disease awareness (25, 33, 42), symptom-management knowledge (6, 21, 24), perceived stress (33), and a repertoire of coping strategies (6, 24); that is, what the family knows and how it appraises the illness before responding. When patients and family members lack knowledge about the disease and worry about the prognosis, effective communication among family members can be undermined, resulting in misunderstandings that increase the patient’s perceived stress, which in turn reduces the level of family resilience and impairs family adaptation (33). The practical enactment of this knowledge and repertoire, including day-to-day symptom management, care problem solving, and acquisition of needed information and services, is itself part of the resilient response, and is therefore classified as an attribute rather than an antecedent (18, 26, 27, 38).

Family Resources. This encompasses the material and psychosocial resources that exist within and around the family before the adaptive response, including the number of family members (30, 34), family type and structure (34, 43), family relationships (34, 41), family functioning (19, 25, 27, 28, 31), family cohesion (6, 31, 34), faith and spirituality (28, 34, 35), economic level (18, 28, 34), and available social support (21, 23, 34). Positive beliefs serve as instruments of emotional support, alleviating stress and anxiety and preserving psychological well-being; patients and family members with faith demonstrate more positive attitudes and coping patterns (35). Lower income and higher healthcare expenditure increase the financial burden (34), whereas stronger family finances help families cope with adverse conditions and reduce the risk of adverse outcomes. Cohesive families possess a strong sense of togetherness and tend to remain positive and weather crises even under adverse conditions (6). Good family functioning reduces the caregiving burden in families of older adults (25); core families with a cohabiting spouse tend to experience heavier burdens, whereas extended families involving other relatives and friends in caregiving perceive lower care pressure and access more social support. Both family and friend networks, together with professional assistance, can significantly enhance family resilience, particularly among vulnerable families (28, 34). Building strong family belief systems, utilizing family resources, developing stable organizational patterns and accessing effective social support can enhance family resilience (19). These resources are antecedents rather than attributes because they constitute the reservoir from which the family draws when enacting resilience.

#### 3.3.2 Consequences

The consequences of family resilience for older patients requiring long-term care refer to the outcomes that follow once the family has mobilized its resilient response to sustained stress. Four consequences were identified: reduced psychological distress and improved mental health, improved quality of care and patient outcomes, enhanced family adaptation and restored functioning, and family growth.

Reduced Psychological Distress and Improved Mental Health. This consequence is experienced primarily by the older adult and family caregivers, and may also extend to other family members. This includes giving meaning to life and care, accepting and coordinating care, reducing negative emotions arising from end-of-life uncertainty, decreasing anxiety and depression, fostering hope for health, and enhancing well-being (21, 23, 24, 36). Positive psychological interventions that create emotional bonding and responsibility can reduce perceived family stress and help the patient and family relieve psychological distress (24).

Improved Quality of Care and Patient Outcomes. By reorganizing family structure and fully utilizing available familial, social and economic resources, resilient families support members in managing internal resources effectively, seeking information actively, and leveraging medical teams and social support, which translates into better symptom management, self-care and treatment adherence for the older patient (25–27). Greater availability and better integration of resources promote the health and development of families and their members (19).

Enhanced Family Adaptation and Restored Functioning. Through strengthened communication, emotional exchange and negotiated responses to emerging needs, family members enhance their organizational and cooperative skills, enabling the family to face problems together and gradually return to normalcy (25, 34, 39). The family rapidly integrates internal and external resources to improve its ability to cope with the care crisis, thereby achieving healthy development (18, 25).

Family Growth: A Rebuilt and Stronger Family. This refers to the state that results from the resilient process: consolidated family beliefs and confidence, enhanced cohesion, acquired caregiving skills and disease knowledge, and improved relationship quality, such that the family emerges stronger than before the adversity (21, 34, 39). In the included concept analysis, the corresponding outcomes are described as acceptance of the situation, changes in life perspectives, enhanced relationship qualities and the reinforcement of resilient properties (29). This resulting state differs from both the pre-existing resource stocks classified as antecedent family resources and the shared beliefs enacted during the process: the family does not merely possess beliefs, cohesion, and support, it has been transformed by mobilizing these resources. By fully utilizing personal and family strengths and social support, the family rediscovers its potential, adapts confidently to adversity, and promotes lifelong learning, social involvement and awareness of healthy living (29).

### 3.4 Distinction from related concepts

#### 3.4.1 Caregiver resilience

Caregiver resilience refers to the individual caregiver’s capacity to withstand and adapt to caregiving stress, and has been conceptually analysed among family caregivers of Asian older adults with dementia (20, 44). Although closely related, caregiver resilience differs from the present concept in its unit of analysis: it locates resilience within an individual, whereas family resilience is a property and process of the family as a whole, in which the older care recipient is a participating member of the resilient unit rather than merely a source of stress. Interventions targeting the two concepts therefore also differ, focusing on individual coping resources in the former and on family-level beliefs, organization and interaction in the latter.

#### 3.4.2 Family resilience in dementia and chronic illness

Family resilience has been examined in dementia (22, 40, 45) and other chronic illness contexts (16, 17), and these conceptualizations overlap substantially with the present one. The long-term care context of older adults, however, adds distinguishing features: care needs are prolonged and typically progressive rather than episodic; functional dependency deepens over time; caregiving is predominantly intergenerational; and the cumulative economic and emotional burden is borne by the family system over years. The present concept therefore specifies family resilience for a sustained caregiving trajectory rather than for a single illness crisis.

#### 3.4.3 Family functioning

Both family resilience and family functioning treat the family as a unit and emphasize family communication, and therefore overlap to some degree. Chinese scholars tend to emphasize the emotional significance of family functioning, viewing it as the capacity to meet the developmental needs of family members, such as mutual support and emotional communication (46). Family functioning represents the functions that develop naturally after family formation, encompassing economic, educational and caregiving functions, whereas family resilience highlights the proactive adjustment, positive communication and joint stress management of all family members, including the patient, in response to pressure and challenges. In the present analysis, family functioning is positioned as an antecedent resource: good family functioning is a protective factor that facilitates family resilience, and under stress, functioning is maintained by mobilizing the resources in family resilience.

#### 3.4.4 Family coping

Family coping refers to the family’s ability to adapt to stressful events and can be divided into positive and negative categories (22). Family resilience emphasizes that, when a family copes with stress, it integrates internal and external resources, utilizes family strengths, exhibits positive developmental characteristics, and seeks to restore balance and harmony (17). Coping strategies available to the family constitute an antecedent resource for family resilience; families with higher resilience typically enact more positive coping strategies, enabling them to deal with adversity and to develop new opportunities from it.

### 3.5 Model case

Following Walker and Avant, the model, borderline and contrary cases below are hypothetical composite cases constructed from the synthesized findings of the included literature; they are not based on empirical participant data, and this study involved no human participants. A model case is a real-world-like example that demonstrates all of the defining attributes of the concept simultaneously and unambiguously.

A 70-year-old man lives with his youngest son in a family of five. Diagnosed with heart failure and having undergone coronary artery bypass grafting 6 years earlier, he requires long-term care. His son and daughter-in-law adjusted their work arrangements to share caregiving (adaptive family organization; intergenerational support), viewing filial piety as a vital element of life (shared positive beliefs); the other three children visit regularly and take turns providing care. The family proactively consults the family doctor to understand the disease (information seeking; proactive engagement with external resources). As the older man’s condition improved, he began participating in community activities, sharing his experience with peers and relishing his ability to help others (sense of self-efficacy; positive appraisal). Concerned about treatment costs, the older man openly raised his worries at a weekly family meeting (open communication; emotional expression). The family reviewed their budget together, asked the physician whether equally effective but more affordable treatment options were available without altering the prescribed regimen, and applied for the financial support scheme for chronic and serious diseases (collaborative problem solving; leveraging social resources). Family members convene weekly to address emerging challenges (collective problem solving). The older man feels profound gratitude for his family’s devotion (positive experience) and hopes to live longer and witness their harmonious, joyful lives. The family’s mutual care and active management of adversity reflect their shared belief that, whatever challenges arise, they can cope with them together.

### 3.6 Borderline case

A borderline case contains some, but not all, of the defining attributes and thereby clarifies the conceptual boundaries of family resilience.

A 68-year-old woman with post-stroke hemiplegia requiring long-term care lives with her 72-year-old husband; their only daughter works in another city. The couple appraises the situation positively, believing that “as long as we face it together, things will get better” (positive cognitive appraisal present), and they communicate openly about their feelings during daily care (supportive interaction partially present). However, the husband alone performs nearly all caregiving tasks; the family has not reallocated roles or sought help from the daughter beyond occasional phone calls (adaptive reorganization largely absent). Unfamiliar with available services, the family has never contacted community home-care or respite services and rarely interacts with neighbours (proactive engagement with external resources absent). The family manages day to day, but the husband’s exhaustion is accumulating and the family’s adaptation remains fragile. This case illustrates that family resilience is a matter of degree: the presence of positive beliefs and open communication alone, without organizational adaptation and engagement with external resources, is insufficient for full family resilience and leaves the family at risk of breakdown under sustained long-term care demands.

### 3.7 Contrary case

A contrary case is a clear example of what the concept is not, demonstrating the absence of the defining attributes.

A 65-year-old woman lives in a family of four. Diagnosed with chronic nephritis and reporting a history of gastritis, she has chosen to remain bed-bound after repeated exacerbations of nephritis—characterized by vomiting and back pain—and unsatisfactory hospital treatment, refusing further hospital care (negative coping; absence of positive appraisal and collaborative problem solving). Feeling hopeless, she cries daily and harbours a fear of dying (negative emotions). Her daughter-in-law, unable to care for her single-handedly, struggles with extensive household duties and chronic back pain, resulting in sleepless nights and occasional outbursts of anger toward the older woman (strained interaction; lack of understanding). The other children, employed in distant cities, neither finance hospital visits (lack of financial support) nor communicate regularly (refusal to confront the situation jointly; absence of adaptive organization). The older woman perceives a loss of social standing (negative experience) and avoids interaction with neighbours and social workers (absence of engagement with external resources). “Talking is pointless; my illness is too severe—what could they possibly do to help? It might as well go unsaid” (refusal to communicate). Other family members visit in tears, exhausted, and are contemplating moving the older woman to a nursing home. This case demonstrates the concurrent absence of all four defining attributes: positive cognitive appraisal, adaptive organization and collaborative problem solving, supportive intergenerational interaction, and proactive engagement with external resources.

### 3.8 Definition of family resilience for older patients requiring long-term care

Drawing on the results of this concept analysis, family resilience for older patients requiring long-term care is defined as a dynamic, recursive family-level process through which the family, when confronting sustained illness and caregiving demands, appraises adversity positively through shared beliefs, adaptively reorganizes roles and solves problems collaboratively, sustains supportive intergenerational interaction, and proactively engages external resources, thereby reducing psychological distress, maintaining the quality of care and family adaptation, and being transformed into a stronger and more competent family unit. This definition is aligned with the final classification in Table 2: appraisal, reorganization, interaction and resource engagement are the enacted constituents of the concept (attributes); health status, socio-demographic characteristics, disease cognition and resource stocks precede it (antecedents); and reduced distress, better care, restored adaptation and family growth follow from it (consequences). The conceptual model including the antecedents, defining attributes and consequences of family resilience for older patients requiring long-term care is shown in Figure 2.

**Figure 2:**
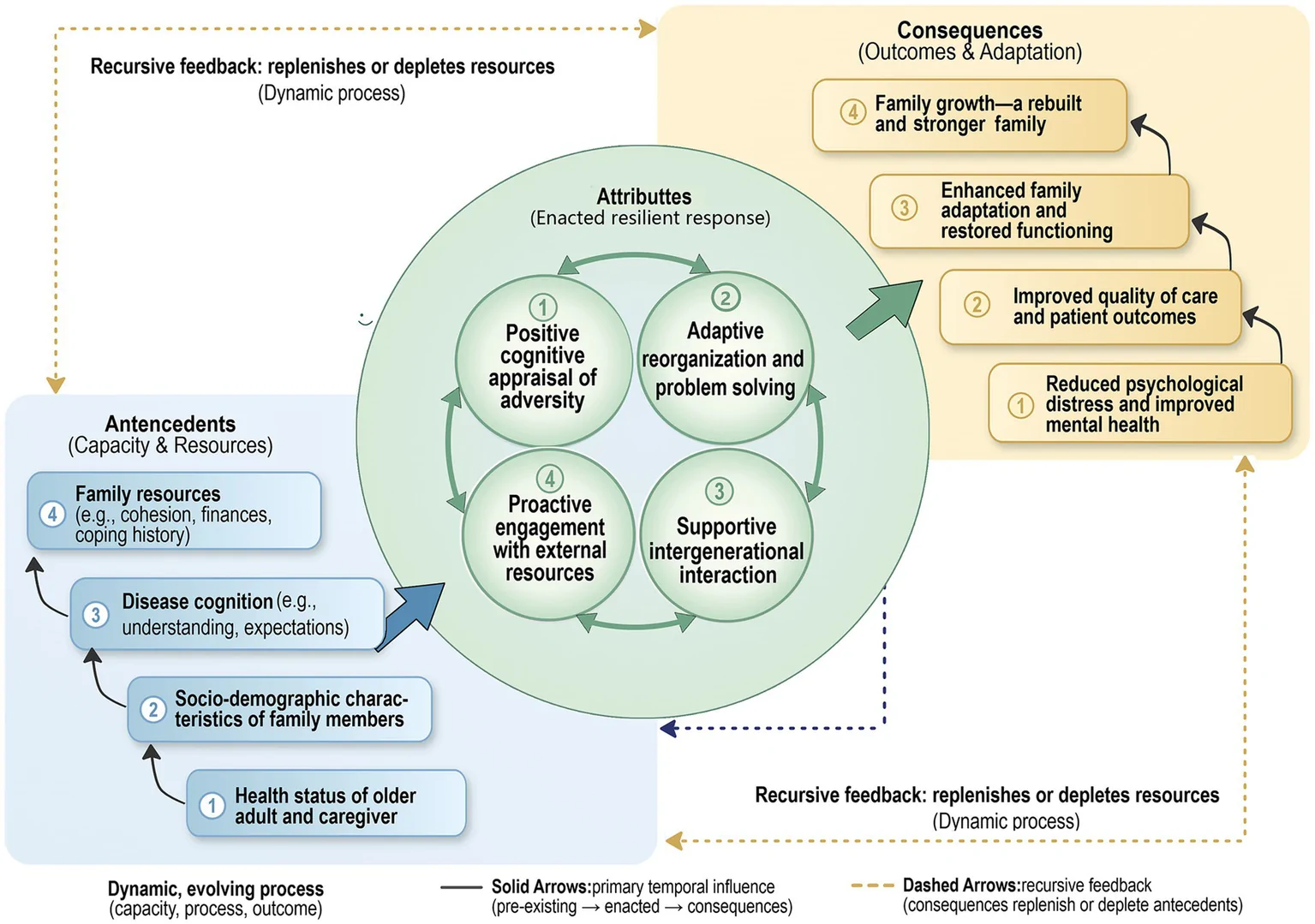
Conceptual model of family resilience for older patients requiring long-term care, including antecedents, defining attributes and consequences.

Family resilience is understood not as a linear sequence but as a recursive, dynamic process that combines elements of capacity, process and outcome. The solid arrows represent the primary temporal direction of influence, from pre-existing antecedent conditions, through the family’s enacted resilient response (attributes), to its consequences. The dashed feedback arrow represents the recursive loop through which consequences in turn replenish or deplete antecedent resources: a family that emerges stronger, less distressed and better adapted enters the next phase of long-term care with replenished cohesion, finances and confidence, whereas a family whose resources are exhausted enters it with diminished antecedents. This theorization resolves the apparent tension between the analytic distinction of antecedents and consequences and the dynamic, evolving nature of resilience over the long-term care trajectory (37, 40).

### 3.9 Empirical referents

Empirical referents are classes or categories of actual phenomena that, by their existence, demonstrate the occurrence of the concept (10). Four instruments currently used to measure family resilience were examined against the four defining attributes identified in this analysis (Table 4).

**Table 4:** Mapping of existing family resilience instruments onto the four defining attributes.

Instrument (reference)	Validation population	A1. Positive cognitive appraisal	A2. Adaptive reorganization and problem solving	A3. Supportive intergenerational interaction	A4. Proactive engagement with external resources
WFRQ/WFRQ-C (47, 49, 50)	General community samples; chronically ill patients and families (Italian version); Thai caregivers of chronically ill older adults	Family belief system (✓)	Organizational communication / problem solving (✓)	Family communication, partial—no explicit intergenerational content (○)	Use of external resources (✓)
WFRQ-CE (43)	566 community-dwelling older adults with disabilities and their caregivers (China)	Family Beliefs (✓)	Organization and Problem Solving (✓)	Family Communication, partial—no explicit intergenerational content (○)	Utilization of Outside Resources (✓)
FRAS (41, 51, 52)	General samples; parents of chronically ill children (Chinese versions)	Maintaining a positive outlook; making meaning of adversity (✓)	Family communication and problem solving (✓)	Family connectedness, partial (○); family spirituality corresponds to an antecedent resource	Utilizing social and economic resources (✓)
FRS (39, 53)	Secondary school students (original); disabled older adults (Chinese adaptation, further testing needed)	Family cognition and perception; forward-looking attitude (✓)	Family management and organization; problem solving (✓)	Intimate harmony; emotional sharing; clear communication (✓)	Social support; family endowment utilization (✓)
FHI (54, 55)	General family samples; patients with moderate-to-severe Alzheimer’s disease (Chinese version)	Commitment, control and challenge beliefs (✓)	Control dimension, partial (○)	Not covered (—)	Not covered (—)

✓ = dimension(s) substantially cover the attribute; ○ = partial coverage; — = not covered. Instrument-source references are cited as conceptual or measurement sources; validation studies cited in this table are among the 30 included studies (Table 2).

#### 3.9.1 Walsh family resilience questionnaire (WFRQ)

The WFRQ was developed by Walsh in 2016 based on her conceptual model (47) and consists of 31 items across three dimensions, plus one open-ended question. Rocchi et al. (48) developed an Italian short version (Walsh-IT) in 2017 for chronically ill patients and their families. Wang and Lu developed the Chinese version (WFRQ-C) among community residents; it contains 26 items across three dimensions—family belief system, organizational communication, and utilization of external resources—with a total-scale Cronbach’s α of 0.97 and a community norm score of 97.00 ± 17.94 (49). The Walsh Family Resilience Questionnaire for Community-Dwelling Older Adults with Disabilities (WFRQ-CE) was validated among 566 dyads of community-dwelling older adults with disabilities and their caregivers in China (43). It consists of 26 items across four dimensions—Family Beliefs, Organization and Problem Solving, Family Communication, and Utilization of Outside Resources, with a total-scale Cronbach’s α of 0.93 and test–retest reliability of 0.96. A Thai validation study among 224 family caregivers of chronically ill older adults supported the original third-order, three-domain structure of the WFRQ and demonstrated strong reliability and concurrent validity (50). The WFRQ-CE is currently the most applicable instrument for assessing family resilience among community-dwelling older adults with disabilities.

#### 3.9.2 Family resilience assessment scale (FRAS)

Developed by Sixbey (51) based on Walsh’s conceptual model, the FRAS includes 54 items and 6 dimensions: family communication and problem solving, socio-economic resource utilization, maintaining a positive attitude, family connectedness, family spirituality, and giving meaning to adversity. The Chinese-adapted scale was tested among 561 parents of chronically ill children, with a total Cronbach’s α of 0.96 (52). The shortened Chinese version includes three dimensions—family communication and problem solving, use of social resources, and holding a positive view—totalling 32 items, with an internal consistency coefficient of 0.96 (41).

#### 3.9.3 Family resilience scale (FRS)

Compiled by Dai (53), the FRS includes 49 items across ten dimensions, such as interpreting adversity, forward-looking attitude, problem solving, intimate harmony, social support, orderliness, emotional sharing, clear communication and collaborative coordination, organized into two subscales: Family Beliefs and Family Strength. Zhang and Li (39) used the FRS to measure the family resilience of disabled older adults, retaining four aspects (family cognition and perception, family management and organization, family resources and utilization, family communication and problem solving) and seven dimensions, totalling 33 items, with a Cronbach’s α of 0.949.

#### 3.9.4 Family hardiness inventory (FHI)

McCubbin and McCubbin (54) developed the Family Regeneration and Adaptation Index (FRAIS), within which the FHI served as a subscale for assessing family resilience. The FHI comprises 20 items in three dimensions: commitment, control and challenge. The commitment dimension gauges family members’ attitudes toward stressful events, the control dimension assesses action-oriented beliefs, and the challenge dimension evaluates positive perceptions of stress. Liu et al. (55) applied the Chinese version to measure family resilience in patients with moderate to severe Alzheimer’s disease, reporting a Cronbach’s α of 0.814.

Mapping existing instruments against the defining attributes outlined in this analysis (Table 4) reveals that coverage varies across measures, with each capturing a subset of the proposed attributes. The WFRQ-CE is the closest match (its four dimensions correspond broadly to the four attributes and it was validated in community-dwelling disabled older adults and their caregivers), yet even it does not explicitly capture the intergenerational content of family interaction; the FRAS was validated mainly in general samples and parents of chronically ill children, and its spirituality dimension corresponds to an antecedent resource in our framework; the FRS was originally developed for secondary school students; and the FHI covers mainly appraisal beliefs. Psychometric evidence for these instruments was established largely in community samples, family caregivers, parents of children with chronic illnesses, or specific diagnostic groups; such evidence should not automatically be treated as validation for older adults requiring long-term care and their families. Scales currently used to measure family resilience in the field of aging have therefore not been adequately tested in older populations with chronic disease requiring long-term care, and their items do not fully capture the family characteristics of this population in the aging-in-place model.

## 4 Discussion

With the acceleration of population aging and increasing life expectancy, the demand for long-term care for older adults is growing, accentuating the physical and psychological burdens on older patients and their families. Promptly identifying family problems and implementing targeted interventions can significantly improve the emotional well-being and health of patients and their families, ultimately enhancing family quality of life. The degree of family resilience reflects a family’s ability to manage long-term stress and its outcomes; identifying and enhancing family resilience is therefore critical for families of older patients. Using concept analysis, this article delineates the antecedents, consequences and defining attributes of family resilience, aiding the harmonization of understanding among researchers, fostering communication, and enabling information sharing.

### 4.1 From a contextualized concept to a theoretical model

The clarity of concepts is foundational to research, and a concept may evolve as understanding deepens, requiring regular revisiting and refinement of its conceptualization (10). The principal contribution of the present analysis is the specification of family resilience as a distinct family-level construct in the long-term care of older adults—complementing prior analyses of individual caregiver resilience (20), a qualitative meta-synthesis of family resilience in long-term care (56), and qualitative study of families caring for disabled older adults (18), as well as analyses of family resilience in cancer and general chronic illness populations (16, 17). Intergenerational support, an important component of family resilience for older patients, has been mentioned by some researchers but not fully explored in previous studies. The content and expression of family resilience vary across cultures, regions and stressful contexts. In China, shaped in part—though not uniformly—by filial norms, many families place particular emphasis on intergenerational mutual support, and older adults often rely heavily on the family as their self-care ability declines; at the same time, considerable variation exists within both Chinese and Western societies, and filial expectations coexist with increasingly diverse family forms and individual preferences (3, 56). Existing theories of family resilience originate mainly in Western countries and have been introduced, adapted and applied by researchers in China, but encounter various problems when applied directly to practice; for example, the localization study of the core concepts of the resiliency model of family adjustment and adaptation (57) notes that the relative weight of individual independence and collective family obligation differs across cultural contexts. In future research, qualitative studies are needed to explore in depth how families of older patients adapt to the pressures of long-term care in the context of strong family networks and intergenerational support in East Asia, so as to develop localized conceptualizations and operational definitions that accurately depict the characteristics of the concept.

### 4.2 Universal and culturally patterned elements

The present analysis suggests that the four defining attributes are likely to represent a universal core of family resilience: positive appraisal of adversity, adaptive organization and joint problem solving, supportive intra-family interaction, and engagement with external resources recur across Western and East Asian literature alike (6, 22, 29, 40, 58). What appears culturally patterned is the content and relative weight of these attributes rather than their presence. In Chinese and other East Asian families, supportive interaction is predominantly organized intergenerationally—adult children caring for aging parents—and is infused with norms of filial responsibility; family beliefs often emphasize collective endurance and family honour; and help-seeking outside the family may be delayed because strong family networks are expected to absorb stress internally (3, 35). In many Western contexts, interaction may be organized more around the spousal dyad and formal services may be engaged earlier (22, 48). These are matters of emphasis rather than categorical differences, and substantial within-culture variation should be acknowledged. Measurement tools and interventions should therefore retain the universal core while adapting items and content to culturally specific expressions.

### 4.3 Public mental health implications

Family caregivers of older adults requiring long-term care show elevated rates of depression, anxiety and chronic stress, and caregiving burden is unequally distributed—concentrated among women, rural families and households with fewer socioeconomic resources (5, 8, 9, 59). A three-level meta-analysis confirmed a significant negative association between family resilience and perceived stress, most pronounced among older adults (60). Because family resilience operates at the level of the family system, it offers a unit of intervention that individual-focused approaches miss: strengthening shared beliefs, intergenerational communication and engagement with community resources may simultaneously protect caregiver mental health and sustain care quality for the older adult (9, 25). At the community level, the attribute of proactive engagement with external resources points to actionable leverage—community-based long-term care services, respite care, caregiver support groups and psychosocial resources can be deliberately linked to families, countering the social isolation that both older adults and their caregivers frequently experience (23, 35). At the policy level, the concept supports family-centered components within long-term care insurance pilots and healthy-aging strategies: routine family resilience assessment could help identify high-risk families for preventive psychosocial support before caregiver mental health deteriorates (2, 11). Positioning family resilience as a family-level protective factor thus connects geriatric care to broader agendas of mental health prevention, equity in the distribution of care burden, and aging and mental health policy.

### 4.4 Tailored assessment tools are needed

Measurement approaches for family resilience in long-term care of older adults need to be grounded in clearly defined attributes to enhance the understanding, measurement and evaluation of family resilience levels. Most existing scales have not been validated among older adults with chronic disease requiring long-term care; although Chinese scales are available, the self-administered versions require further testing in other long-term care populations (39). Future work can integrate the present definition and attribute structure with localized structural models to develop specific assessment tools for this population. Researchers can use the operational definition proposed here to analyse whether existing instruments contain the defining attributes and to select appropriate instruments in relation to the purpose of the study, thereby improving the reliability of measured family resilience levels.

### 4.5 Family resilience interventions are needed but few

The proposition of family resilience indicates that practitioners can improve patient outcomes by focusing on both individuals and their families, but how to effectively tap the potential of the patient’s family and implement interventions to improve family resilience remains a difficult problem. Only one of the included studies evaluated a family-centered nursing intervention for older patients with long-term care needs; this scarcity may be related to the previous ambiguity of the concept. Although family resilience enhancement programs have been piloted among families of older adults with dementia (32), such interventions remain scarce for older patients requiring long-term care more broadly. Clarifying the definition of family resilience for older patients requiring long-term care provides a theoretical basis for constructing intervention strategies. The definition supports more direct understanding of family resilience by nurses, facilitating the selection of appropriate assessment tools, the early identification of families with low family resilience, and interventions targeting weaknesses in family beliefs, family organization, intergenerational communication and resource engagement to improve family resilience in this population.

## 5 Limitations and future study

Several limitations should be acknowledged. First, only Chinese and English literature were included, which may introduce linguistic and cultural bias. Second, the model, borderline and contrary cases are hypothetical composite cases constructed from the literature rather than derived from empirical participant data; empirical studies are needed to test and refine the proposed attributes. Third, although an explicit temporal-logical criterion was applied, the classification of constructs into antecedents, attributes and consequences inevitably involves interpretive judgement, and alternative classifications are conceivable; the integrated matrix and included-study list in Table 2 are provided to make these judgments transparent and contestable. Future studies may use qualitative methods to explore the connotation and extension of the concept in depth, develop localized conceptualizations and measurement tools, and build theoretical models and interventions for different populations and stress scenarios.

## 6 Implications for practice

By gaining a comprehensive understanding of the process of family resilience for older patients requiring long-term care, nurses and community health practitioners can develop localized theoretical frameworks, research instruments and family-centered interventions. At the public health level, the concept offers a family-level target for mental health promotion and the prevention of caregiver psychological distress within community-based long-term care.

## 7 Conclusion

This study provides an in-depth concept analysis of family resilience for older patients requiring long-term care, identifying four defining attributes—positive cognitive appraisal of adversity, adaptive reorganization and problem solving, supportive intergenerational interaction, and proactive engagement with external resources; four antecedents—health status of the older adult and the caregiver, socio-demographic characteristics of family members, disease cognition, and family resources; and four consequences—reduced psychological distress and improved mental health, improved quality of care and patient outcomes, enhanced family adaptation and restored functioning, and family growth. A recursive, dynamic conceptual framework was formed on this basis, providing a theoretical foundation for localized theoretical models, the development of research tools, and nursing interventions.
