# Supplementary material for: Family resilience for older patients requiring long-term care: a concept analysis
Source: Front Public Health. 2026 Oct 22;14:1962121. doi: 10.3389/fpubh.2026.1962121 (PMC13735106; doi:10.3389/fpubh.2026.1962121)
Supplement: Supplementary file 1 [file Table_1.docx]

**Supplementary Table 1. Database-specific search strategies (Web of Science, Embase)**

| **Database** | **Search strategy** |
| --- | --- |
| Web of Science | TS=(“family resilien*” OR “family strengt*” OR “family adaptation” OR “family coping”) AND TS=(“long-term care” OR “long term care” OR “family caregiv*” OR “informal care*” OR “home care”) AND TS=(older* OR aged OR elderly OR geriatric OR “chronic diseas*” OR disab*); timespan: inception to 15 September 2026; language: English |
| Embase | ('family resilience'/exp OR 'family resilien*':ti,ab OR 'family strengt*':ti,ab OR 'family adaptation':ti,ab OR 'family coping':ti,ab) AND ('long term care'/exp OR 'long-term care':ti,ab OR 'long term care':ti,ab OR 'family caregiv*':ti,ab OR 'informal care*':ti,ab OR 'home care'/exp OR 'home care':ti,ab) AND ('aged'/exp OR older*:ti,ab OR aged:ti,ab OR elderly:ti,ab OR geriatric:ti,ab OR 'chronic disease'/exp OR 'chronic diseas*':ti,ab OR 'disabled person'/exp OR disab*:ti,ab); inception to 15 September 2026; [English]/lim |
