# Supplementary material for: Family resilience for older patients requiring long-term care: a concept analysis
Source: Front Public Health. 2026 Oct 22;14:1962121. doi: 10.3389/fpubh.2026.1962121 (PMC13735106; doi:10.3389/fpubh.2026.1962121)
Supplement: Supplementary file 2 [file Table_2.docx]

**Supplementary Table 2. Source-by-category analytical matrix (category-by-row view)**

| **Category** | **Component and definition in this analysis** | **Supporting included studies(No.,author–year)** |
| --- | --- | --- |
| **Attribute** | A1. Positive cognitive appraisal of adversity. Shared positive beliefs, acceptance, hope, optimism and meaning-making with which the family appraises illness and caregiving | (6) Zhang W 2022; (17) Yan & Li 2021; (18) Abulaiti B 2022; (21) Ye 2021; (22) Deist & Greeff 2017; (23) Sanchez-Teruel 2022; (24) Yan & Zhang 2017; (25) Zhang MM 2022; (26) Lin 2024; (27) Dunbar 2008; (28) Yu 2022; (29) Oh & Chang 2014; (37) Lv 2026 |
|  | A2. Adaptive reorganization and problem solving. Enacted flexibility, role reallocation, coordination and joint problem solving in response to caregiving demands | ((6) Zhang W 2022; (17) Yan & Li 2021; (18) Abulaiti B 2022; (21) Ye 2021; (23) Sanchez-Teruel 2022; (24) Yan & Zhang 2017; (25) Zhang MM 2022; (26) Lin 2024; (27) Dunbar 2008; (28) Yu 2022; (30) Zhou J 2022; (31) Nissen 2016; (32) Kim 2019; (40) Yang & Song 2025 |
|  | A3. Supportive intergenerational interaction. Open communication, emotional expression, mutual care and support among generations in daily caregiving | (6) Zhang W 2022; (18) Abulaiti B 2022; (21) Ye 2021; (23) Sanchez-Teruel 2022; (25) Zhang MM 2022; (26) Lin 2024; (27) Dunbar 2008; (30) Zhou J 2022; (31) Nissen 2016; (32) Kim 2019; (33) Zhang M 2021; (34) Jin 2022; (35) West 2011; (36) Jia 2025; (37) Lv 2026; (40) Yang & Song 2025 |
|  | A4. Proactive engagement with external resources. Active information seeking, use of social support, and access to community and professional services | (17) Yan & Li 2021; (18) Abulaiti B 2022; (21) Ye 2021; (22) Deist & Greeff 2017; (23) Sanchez-Teruel 2022; (26) Lin 2024; (27) Dunbar 2008; (30) Zhou J 2022; (34) Jin 2022; (35) West 2011; (38) Moral-Fernandez 2018 |
| **Antecedent** | B1. Health status of the older adult and the caregiver. Functional and disease-related conditions and caregiving load existing before the family's adaptive response | (5) Muñoz-Cruz 2024; (17) Yan & Li 2021; (18) Abulaiti B 2022; (19) Zhou & Wang 2021; (26) Lin 2024; (30) Zhou J 2022; (31) Nissen 2016; (34) Jin 2022; (37) Lv 2026 |
|  | B2. Socio-demographic characteristics of family members. Age, gender, education level and residential status of family members | (18) Abulaiti B 2022; (24) Yan & Zhang 2017; (25) Zhang MM 2022; (28) Yu 2022; (34) Jin 2022; (36) Jia 2025 |
|  | B3. Disease cognition. Pre-existing disease awareness, symptom-management knowledge, perceived stress and coping repertoire (enacted management is classified under the attributes) | (6) Zhang W 2022; (17) Yan & Li 2021; (18) Abulaiti B 2022; (21) Ye 2021; (22) Deist & Greeff 2017; (24) Yan & Zhang 2017; (25) Zhang MM 2022; (33) Zhang M 2021; (34) Jin 2022; (40) Yang & Song 2025; (42) Smith & Shaw 2017 |
|  | B4. Family resources. Pre-existing family cohesion, functioning, structure/size, economic level, faith and spirituality, and available social support | (6) Zhang W 2022; (17) Yan & Li 2021; (18) Abulaiti B 2022; (19) Zhou & Wang 2021; (21) Ye 2021; (25) Zhang MM 2022; (27) Dunbar 2008; (28) Yu 2022; (30) Zhou J 2022; (31) Nissen 2016; (34) Jin 2022; (35) West 2011; (37) Lv 2026; (40) Yang & Song 2025 |
| **Consequence** | C1. Reduced psychological distress and improved mental health. Decreased anxiety, depression and perceived stress; increased hope and well-being of family members | (17) Yan & Li 2021; (18) Abulaiti B 2022; (21) Ye 2021; (23) Sanchez-Teruel 2022; (24) Yan & Zhang 2017; (36) Jia 2025; (37) Lv 2026 |
|  | C2. Improved quality of care and patient outcomes. Better symptom management, self-care, treatment adherence and quality of life of the older patient | (19) Zhou & Wang 2021; (25) Zhang MM 2022; (26) Lin 2024; (27) Dunbar 2008 |
|  | C3. Enhanced family adaptation and restored functioning. The family reorganizes, integrates resources and gradually returns to normalcy and healthy development | (17) Yan & Li 2021; (18) Abulaiti B 2022; (25) Zhang MM 2022; (32) Kim 2019; (34) Jin 2022; (39) Zhang & Li 2024; (40) Yang & Song 2025 |
|  | C4. Family growth: a rebuilt and stronger family. A resulting transformed state: consolidated confidence, acquired caregiving skills and knowledge, enhanced relationship quality, and a family that emerges stronger than before | (17) Yan & Li 2021; (21) Ye 2021; (29) Oh & Chang 2014; (34) Jin 2022; (39) Zhang & Li 2024; (40) Yang & Song 2025 |

*This matrix presents the same evidence as Table 2 but organized by category: for each antecedent, attribute and consequence, the supporting included studies are listed (reference number and author–year), so that each classification can be traced to the eligible evidence from either the study-by-study view (Table 2) or the category-by-category view (this tab*
